# An Aqueous Composition for Lubricant‐Free, Robust, Slippery, Transparent Coatings on Diverse Substrates

**DOI:** 10.1002/gch2.201700097

**Published:** 2018-01-02

**Authors:** Avijit Baidya, Sarit Kumar Das, Thalappil Pradeep

**Affiliations:** ^1^ DST Unit of Nanoscience (DST UNS) and Thematic Unit of Excellence (TUE) Department of Chemistry Indian Institute of Technology Madras Chennai 600 036 India; ^2^ Department of Mechanical Engineering Indian Institute of Technology Madras Chennai 600036 India

**Keywords:** aqueous suspension, atmospheric water capture, composite solution, liquid repellence, transparent coatings

## Abstract

Transparent, durable coating materials that show excellent liquid repellency, both water and oil, have multiple applications in science and technology. In this perspective, herein, a simple aqueous chemical formulation is developed that provides a transparent slippery coating without any lubricating fluids, on various substrates extended over large areas. The coatings repel liquids having a range of polarity (solvents) as well as viscosity (oils and emulsions) and withstand mechanical strains. Exceptional optical transparency of 99% in the range of 350–900 nm along with high stability even after cyclic temperature, frost, exposure to sunlight, and corrosive liquids like aqua regia treatments, makes this material unique and widens its applicability in different fields. Besides, being a liquid, it can be coated on an array of substrates independent of their underlying topography, by various easily available techniques. Aside from these interesting properties, the coating is demonstrated as a potential solution contributing to the remediation of one of the biggest global issues of tomorrow: affordable drinking water. The coated surface can capture 5 L of water per day per m^2^ at 27 °C when exposed to an atmosphere of 63% relative humidity.

## 1 Introduction

Materials capable of imparting amphiphobic (hydrophobic and oleophobic) coatings are highly desirable for today's varied applications such as touch screen displays to glasses used in buildings, automobiles, etc. and are being intensely researched upon.^1^ Although robustness toward chemical and mechanical stresses is one of the most needed/desired criteria of such coatings, high optical transparency has also drawn much attention of both industries and academia.[[qv: 1h,2]] Liquids usually have a high contact angle (CA) and a low contact angle hysteresis (CAH = advancing angle [θ_A_] − receding angle [θ_H_]) on these surfaces. In some cases, the surface energy of these coatings is so low that it makes liquids to bounce or roll over the surface and sit as a sphere, an effect known as superamphiphobicity.[[qv: 1b,e,3]] While lotus leaf effect is the inspiration behind these developments,^4^ the trapped air in the microstructured surface of lotus leaf is ineffective against liquids with low surface tension.^5^ To address this, recent research has come up with a “re‐entrant surface curvature technology,” a hierarchically developed surface structure with sufficient recess between the surface structure and its base causing liquids to sag below, without coming in contact with its sides or the base.^3^, ^6^ These materials have ultralow CAH and consequently high repellency for oils. Modulating the aspect ratio of these microstructures allowed the construction of surfaces with a static CA close to 160° for hexadecane, although the flat surface was oleophilic.^7^ However, the creation of these microstructures compromises the transparency[[qv: 6d]] of the material due to increased refractive index.[[qv: 1b,7,8]] Moreover, intricate microfabrication technology needed to create such surfaces makes them rather expensive and in addition offer limited surface compatibility.[[qv: 3,6a]] On the other hand, stability or longevity of such surfaces are questionable, although a few reports on robust liquid repelling coatings exist.[[qv: 1h,9]] Furthermore, high CA leading to lower contact area between the liquid drops and these surfaces makes it difficult to accomplish various industrially significant features including heat transfer, condensation, and many others.^10^ In this context, slippery liquid‐infused porous surfaces (SLIPS), with equally efficient liquid repellent property, known to possess low CA and low CAH while allowing high contact area, is an alternative.^5^, ^11^ *Nepenthes pitcher*‐plant inspired surfaces of this kind were developed by infusing low surface tension liquids, such as perflurinated oils, inside the nano/microstructured porous matrices.[[qv: 2b,5,12]] Recently, Chen et al. have reported a cellulose‐based transparent slippery surface that repels both liquids and ice.[[qv: 2b]] Kang et al. have developed transparent hydrophobic electrodes in the context of outdoor solar cell devices where nonwetting property keep the surface clean and allows efficient/effective absorption of sunlight.^13^ Application of these bioinspired surfaces is also known in different fields of science and technology.[[qv: 1d,5,14]] Furthermore, such water repelling surfaces having large contact area of water droplets are more efficient for condensation‐based technologies like water/humidity harvesting that can help to solve the water scarcity. In this context, Kim et al. have developed a graphene‐based hydrophobic surface.^15^ However, large scale production of such surfaces can be an issue and expensive as it was obtained at very high temperatures (≈1000 °C) through in situ chemical‐vapor‐deposition. Therefore, designing a simple coating material to develop an affordable and scalable liquid repelling surface (both for water and for other liquids) devoid of lubricating fluids with durability is important. Incorporation of transparency can also explore the applications of such surfaces toward different global issues including energy crisis.

Several methods have been introduced to create amphiphobic surfaces.[[qv: 1h,16]] Among these, designing coating materials through the sol–gel process is one of the increasingly developing methods and is intensely researched upon because of its reduced complexity (in production) and diverse substrate compatibility.[[qv: 1e,f,17]] However, most of the time, organic solvents, such as ethanol, acetone, hexadecane, dimethylformamide, and tetrahydrofuran, are heavily used that increase the concern related to the production cost and associated environmental problems.[[qv: 6a,16a,18]] Similar problem exists for slippery coating material as well.[[qv: 2b]] A few reports on aqueous coating materials for superhydrophobic surfaces exist.^19^ However, such materials for superamphiphobic or slippry surfaces are not explored much.^20^ Recently Lin and co‐workers have demonstrated a robust superamphiphobic surface by spraying a stable aqueous fluorinated nanoparticle dispersion.[[qv: 1e]] Such waterborne coatings yielding high transparency are desired for various applications. In this perspective, use of polydimethylsiloxane, a widely used hydrophobic coating material, also gets limited attention because of its solvent (organic) and substrate (except glass) compatibility. It also possesses inherent limitation in transparency when exposed to different temperatures. This suggests the necessity to develop a coating material in water that can provide a robust liquid repellent slippery coating over various substrates, irrespective of their shape, size, and surface morphology.

In this work, we present a novel waterborne material which is a liquid at room temperature and shows excellent liquid repellent property (without any lubricating fluids) upon curing over the surface. The material can be painted or coated as a thin film on various substrates, such as metal, glass, hard plastic, and paper, etc., despite their varying surface morphology. Being a liquid, large area coating by processes such as spray coating, spin coating and doctor blading are possible which widen its applicability. Coated substrates show excellent liquid repellency with 99% transparency when compared to clean room treated glass slides. Interestingly, coating withstands various thermo‐mechanochemical damages without any adhesives and retains its properties intact. We believe, a combination of reduced surface energy along with rigid nanoscale structures, which form during the rapid polymerization process, helps these coatings to repel a wide variety of liquids irrespective of their polarity and viscosity. Beside these multiple effective properties, applicability of this coating for efficient water condensation is demonstrated as a proof of concept for atmospheric water capture that can resolve one of the biggest global issues namely, the water crisis.

## 2 Synthesis

In the synthesis protocol, two different functional silanes, perfluorooctyltriethoxysilane (FS) (1.8 vol%) and aminopropylaminoethyltrimethoxysilane (AS) (40 vol%), were added in water and stirred for 6–7 h at room temperature. The final composite was in liquid form and was coated on various substrates by spray coating and was cured in an oven at 110 °C for 2 h. Although spray coating technique was used to prepare the samples, other methods such as spin coating, dip coating, and doctor blading methods may be used to prepare the samples without compromising the transparency of the surface. While the thickness of the coating can be modified depending on the volume of the material used, nearly 100 µL of the as synthesized composite material was sprayed to make a thin film on a surfaces area of 75 × 26 mm^2^. To demonstrate the wide applicability of this material, different types of substrates were coated and tested for material compatibility and mechano‐thermochemical stability. These are explained and demonstrated later in the text. Although AS is known to be nontoxic in biological experiments,^21^ and C—F bonds of FS are stable, the formulation can be used with caution.

## 3 Results and Discussion

**Figure 1:**
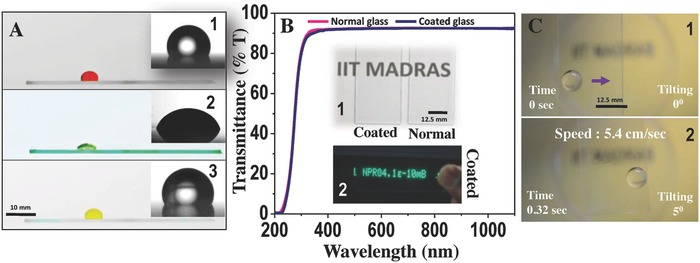
A) Photographs of the coated surface with different liquids; 1‐water, 2‐toluene, 3‐aqua regia. Inset: The static contact angle of the respective liquids. B) Percentage transmission of coated glass in comparison to a normal glass. The coated surface showed 99% transmission when compared to the normal uncoated surface. 1. Coated surface just before the written letters. 2. Electronic letters through the coated surface (distance: 8–9 cm approximately). C) Image shows that at low tilting angle (5°) of the coated surface water rolled off at a speed of 5.4 cm s^−1^.

**Figure** **1**A demonstrates that a wide variety of liquids from water to toluene and even corrosive acids such as aqua regia sit over the coated surface without spreading. This indicates low surface free energy of the coating which was investigated further. Inset shows the static CA of those respective fluids over the surface. Exceptionally high optical transparency of the coated glass was observed using UV–vis spectrometry (Figure 1B). About 99% transmission in comparison to clean room‐treated glass is shown in Figure 1B, Insets 1 and 2, and Video S1 in the Supporting Information. No observable differences in the visibility were found even when the coated surfaces were tested in front of an electronic display, ≈8–10 cm away from the surface (Figure 1B, Inset 2). Initially, the wetting property of the coated surface was tested with the movement of the water drop on the surface when it was tilted manually for a few degrees (details are in the Experimental Section and the Supporting Information). The velocity of the slipped water drop was measured as 5.4 cm s^−1^ (Figure 1C and Video S2, Supporting Information). Such a property of the coating resembles the slippery surfaces and was further studied in detail later on. This phenomenon can lead to a range of applications in energy reduction, in the context of liquid transport.

**Figure 2:**
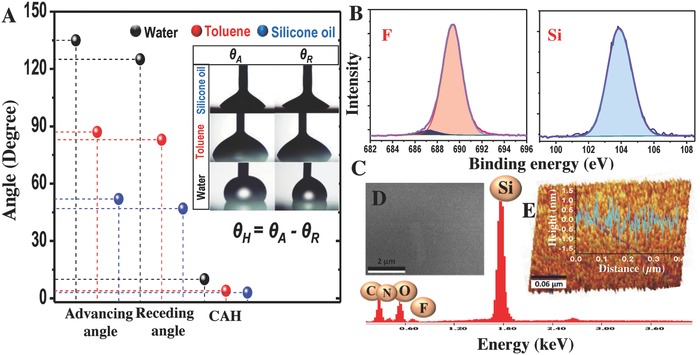
A) The advancing angle (θ_A_), receding angle (θ_R_), and contact angle hysteresis (θ_H_) of water, toluene, and silicone oil, respectively. Inset shows the corresponding images. Physicochemical properties of the coated substrate. B) The XPS spectra of the coated surface showing fluorine 1s and silicon 2p region. C) EDAX spectrum shows the elemental composition of the coating. D) SEM image reveals the absence of micrometer scale morphology. E) AFM image and the average roughness of the coating, which is in nm scale.

Low surface tension liquids (such as oils and emulsions) usually wet the surface and spread easily. Here, the wetting behaviors of the coated surface toward various liquids having different surface tensions were assessed by both static and dynamic CA measurements where dynamic CA is represented in terms of CAH. For instance, water droplet placed on the coated surface formed a static contact angle of 134° ± 2°; whereas, for toluene and silicone oil, it was 86° ± 2° and 52° ± 2°, respectively (**Figure** **2**A). It correlates with the surface tensions of the respective liquids. However, low CAH in the range of 10° ± 2°, 4° ± 2°, and 3° ± 2° for water, toluene, and silicone oil, respectively, reveals the extent of the slippery nature of the coating toward different liquids despite having low static contact angle on the coated surface (Figure 2A). Inset shows a picture of advancing and receding angle of the respective fluids. Surface structure and the chemical composition, being the underlying reasons of the wetting property, the coated surface was characterized in detail through various spectroscopic and microscopic techniques. X‐ray photoelectron spectroscopy (XPS) spectra reveal that the coating is largely composed of silica and fluorocarbons (Figure 2B). Peak at in the region of 103.5 eV corresponds to the deconvoluted Si 3p peak of Si^4+^ which matches exactly with that of silica (SiO_2_). The presence of silicon, nitrogen, and other elements along with fluorine was proved from the energy dispersive analysis of X‐rays (EDAX) spectrum and mapping as well (Figure 2C and Figure S1, Supporting Information). Wide area powder X‐ray diffraction of the film showed an amorphous background, similar to the glass substrate (Figure S2, Supporting Information). This was also observed in the qualitative elemental distribution of silicon and oxygen (1:2) in the EDAX mapping of the surface (Figure S1, Supporting Information). This detailed chemical characterization concludes that the backbone of the coating is made of silica network. This was also reflected in the robustness of the coating toward thermo‐mechanochemical perturbation, explained later on. Though scanning electron microscopy (SEM) reveals the absence of micrometer scale structure (Figure 2D), atomic force microscopy (AFM) imaging confirms that the roughness of the coatings is very low, less than 1 nm (Figure 2E). Both of these studies (spectroscopic and microscopic) suggest that the nanostructuring and the presence of appropriate functionalization are the reasons of this observed wetting property. To demonstrate the extent of water repellency of the coating material a glass slide was coated in the shape of ‘’ consisting of two uncoated patches in the middle. The hydrophobic coating in the periphery of the glass slides acted as an invisible barrier to contain ≈4 mL of water in the uncoated hydrophilic patches having a surface area of 7.8 cm^2^. As a result of this confinement, the water could reach a height of 0.4 cm in each of the patches (Figure S3 and Videos S3 and S4, Supporting Information). This concept of the invisible barrier has been used by others too and can be used in trapping, directing as well as retaining water and can have immediate applications in biological and environmental areas.^22^

**Figure 3:**
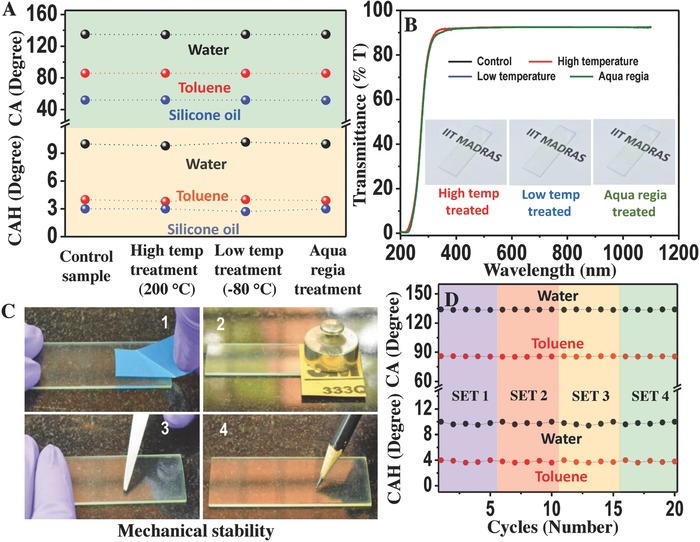
A) Stability of the coating upon high temperature (200 °C), low temperature (−80 °C), and aqua regia treatments. CA and CAH of the liquids (water, toluene, and silicone oil) on the treated surfaces compared to the control. B) Corresponding percentage transmission of the treated substrates shows no change in comparison to the control. Inset: Pictorial representation of treated surfaces. C) Test for mechanical robustness. 1) Peeling‐off experiment, 2) sand paper abrasion, 3) knife scratch, and 4) reusability measurements using write and erase experiments. D) Durability test in cyclic fashion. Sets 1 and 2: Treating at high temperature (200 °C) and low temperature (−80 °C). Set 3: Effect of chemicals (surface was dipped inside different organic solvents, oil and emulsion). Set 4: Direct exposure to sunlight.

**Figure 4:**
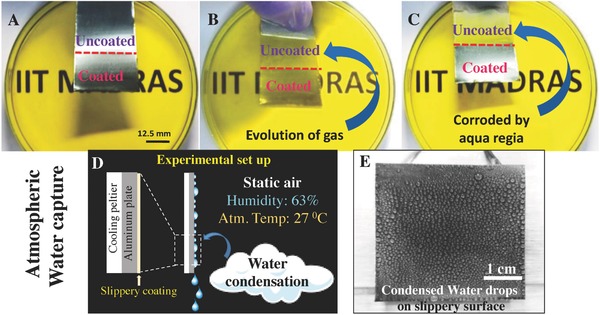
Properties of the coated surface. A). Response of the surface to aqua regia treatment. The metal surface (stainless steel) was dipped into aqua regia. B) There was an evolution of hydrogen gas from the uncoated part and C) it eventually corroded, while the coated region remained intact. D) Schematic representation of atmospheric water capture. E) Real time experimental setup with condensed water drops over the coated surface where surface temperature was cooled down to 8 °C by a peltier cooling system. The environmental temperature was 27 °C with 63% relative humidity.

For applications in touch screens, goggles, and windscreens, the coating needs to be resistant to acute temperature fluctuations and chemical disruptions without compromising transparency of the surface. The stability and wettability of the coating against thermal and chemical damages were evaluated with extreme temperatures (high and low) and aqua regia (corrosive acid mixture). For all the cases, water, toluene, and silicone oil were used to study the wettability of the treated surfaces in terms of static CA and CAH. Glass substrates were assessed primarily to monitor the transparency and integrity of the coating. For high temperature treatment, surface was annealed at 200 °C for 4 h and it was observed to retain its surface free energy intact compared to the control sample (coated slides at room temperature). This was reflected in the CA and CAH of the liquids over the treated surface (**Figure** **3**A and Video S5, Supporting Information). The stability of the coating at low temperatures was tested by incubating the surface at −80 °C for 8 h. In this case, a similar liquid repellent property was observed for the treated surface (Figure 3A and Video S6, Supporting Information). Chemical robustness of the material was tested by incubating the coated glass in aqua regia for 10 min. Interestingly, the wettability of the coating remained unaltered and the surface functioned properly (Figure 3A and Video S7, Supporting Information). Optical transparency of all the treated surfaces was also found to remain unchanged from that of the control (Figure 3B). Inset of the figure pictorially represents the treated surfaces. These seem to be highly advantageous for places where frost formation on windshields is a serious concern. To be used as a nonwettable coating material for day to day use, mechanical stability is a mandatory compliance. This was assessed by knife scratch, peeling off, and abrasion tests. These tests were done using scissor, scotch tape, and a sand paper (keeping a load of 50 g on the sand paper) (Figure 3C, 1–3). For all the cases, even after 20 complete cycles, the coatings remained pristine with uncompromised wetting behavior toward different liquids (Figure S4, Supporting Information). Transparency of the treated surfaces also remained intact although there was some sign of knife scratches on the particular surface (Figure S5, Supporting Information). Reusability as well as stability of the coating was further evaluated by write and erase tests (Figure 3C, 4) where pencil streaks were easily erasable without damaging the unique properties of the coating. In this case also treated surface was also checked for wettability and transparency (Figures S4 and S5, Supporting Information).

Longstanding durability of the coating to different cyclic perturbations was studied by subjecting the sample to consecutive cycles of different sets of conditions such as high temperature, frost, chemical treatment (organic solvents, oil and emulsion), and exposure to sunlight. Change in the wettability was measured (CA and CAH) after each and every cycle of different sets (details in the Experimental Section and the Supporting Information), which shows a constant value of 134° ± 2° and 86° ± 2° for CA and 10° ± 2° and 4° ± 2° for CAH on an average for water and toluene, respectively. These values remained unaltered even after four different sets of experiments (total of 20 cycles) (Figure 3D).

Application of such materials is not confined to glass substrates alone. To explore the universality or compatibility of the material with different substrates, this material was applied to a wide variety of substrates starting from metal to wood and plastic (Figure S6, Supporting Information). Consistency in the physical appearance of the original substrates even after the coating provides an added advantage (Figure S7, Supporting Information). All the substrates (coated glass and wood surface shown here) showed excellent resistance to wetting by nonpolar fluids such as oil and oil‐water emulsion (Figure S8 and Video S8, Supporting Information). Stability of the coatings on metal substrates was evaluated by treating them with the corrosive acid, aqua regia. The metal surface coated with the newly synthesized material remained unaffected (**Figure** **4**A–C and Video S9, Supporting Information) while uncoated surface changed its color immediately with the evolution of hydrogen gas.

Figure 4D schematically demonstrates effective application of such slippery coatings in real life. Affordable drinking water being a global issue to concern, atmospheric water capture has become a hot topic of research. This needs efficient condensation of humidity and transportation of the droplet formed on the surface. In this context, low hysteresis and low contact angle (high contact area) surfaces having excellent durability can be a good solution as water condenses over such surfaces easily. Figure 4E demonstrates a proof of concept experiment. Humidity and temperature are the governing parameters for this phenomenon. At 63% humidity and 27 °C, our coated surface enables condensation of 5 L of water per m^2^ in a day. Here, a peltier cooling system was used to cool the surface down to 8 °C. We believe that the efficiency of such water collection can be maximized by patterning the surface. We note that overall collection efficiency is not only an issue of efficient condensation but also transport which is mostly controlled by the wettability of the surface as well as CAH.

High liquid repellent nature of the coating originates from the presence of low surface energy molecules as well as the nanostructures formed in situ. While efficient adhesion property of silanes on the surface of various substrates makes this coating universal, the formation of amorphous silicate structure upon curing, which is inert toward a range of chemicals including strong acids, makes this material robust toward various mechanical and chemical perturbations. Stability and high transparency of the coating at varying temperatures also can be explained easily from the physical and the chemical structure of the material, which are similar to silicate glass.

## 4 Conclusion

In conclusion, a waterborne, easy to synthesize, robust coating material has been formulated that shows high liquid repellency, without the use of any lubricating fluids. The coating showed excellent stability toward mechanical strains with uncompromised optical transparency. Transparency as well as liquid‐repellent properties of the coating was maintained even after extreme thermochemical treatments. Being a water‐based liquid material, it enables the creation of large surface area slippery surfaces with a simple coating procedure and decreases environmental concerns and risk of organic solvents at the same time. While extreme repellency toward a wide variety of liquids can widen its industrial use by minimizing transportation cost of fluids through pipelines, transparency in extreme conditions along with other properties can provide easy solutions for display and automobile industries. Beside these, application of this surface toward solving one of the biggest global issues, namely affordable clean/drinking water, is demonstrated as a proof of concept.

## 5 Experimental Section

*Contact Angle Measurements with Cyclic Thermo‐Mechanochemical Perturbation*: To measure the durability of the material, the coated surface was tested with cyclic thermo‐mechanochemical perturbations. For all experiments (Sets 1–4), the same surface was used repeatedly. Change in wettability after each cycle of every set was tested with static CA and CAH measurements. Here, water and toluene were used as test liquids. To test thermal stability, the coated surface was treated at high (200 °C, Set 1) and low temperatures (−80 °C, Set 2), respectively for 5 h in every cycle. This was repeated for five times for both the cases. For assessing chemical inertness, the coated surface was dipped inside the solvent and kept for 2 h. Various polar and nonpolar solvents such as ethanol, Tetrahydrofuran (THF), Dimethylformamide (DMF), hexane, silicone oil, and an emulsion (a mixture of paraffin oil and water) were used to simulate chemical strain. To quantify the effect of direct sunlight, coated surfaces were exposed to sunlight and checked at regular time intervals of 8 h for a duration of 40 h.

*Tilting Angle Experiment*: To measure the extent of slipperiness, movement of a water droplet upon tilting the slip surface was captured by camera and its velocity was calculated, which is directly related to the friction or slipperiness of the coated surface. The motion was induced by tilting the surface manually.

*Chemicals*: All the chemicals were commercially available and were used without further purification. FS was purchased from Aldrich. AS was purchased from Rishichem Distributors. Ethanol, THF, DMF, hexane, and silicon oil (Analytical Reagents (AR) grade) were procured from RANKEM, India. Sand paper (P320) was purchased from a local hardware shop. Peltier cooling system was purchased from a local electronics shop.

*Instrumentation*: UV–vis absorption/extinction spectra were recorded using a Perkin‐Elmer Lambda 25 spectrophotometer in the range of 200–1100 nm using absorption cells having a path length of 1 cm.

AFM imaging was done with Witec Alpha300 S confocal Raman spectrometer with an AFM attachment (Zeiss 20x objective). AFM imaging was carried out in noncontact mode with a cantilever of following parameters: thickness 4 µm, length 125 µm, width 30 µm, resonance frequency 320 kHz, and force constant 42 N m^−1^.

Electron microscopy imaging was done using an FEI Quanta 200 environmental scanning electron microscope with EDAX energy dispersive spectroscopy (EDS) system, to study the surface morphology of the coated substrates.

XPS measurements were carried out using an Omicron electron spectroscopy for chemical analysis (ESCA) Probe spectrometer with polychromatic Mg *K*_α_ X‐rays (1253.6 eV). The X‐ray power applied was 300 W. The pass energy was 50 eV for survey scans and 20 eV for the specific regions. The sample solution was spotted on stainless steel XPS sample plates and dried in vacuum. The base pressure of the instrument was 5.0 × 10^−10^ mbar. The binding energy was calibrated with respect to adventitious C1s feature at 285 eV.

Contact angle and CAH of liquid droplets (water, toluene, and silicone oil) on the different coated substrates were measured using a Holmarc contact angle meter.

Nikon D5100 camera was used to capture all the pictures and videos.

Spray of the water dispersed material was performed with Badger Air‐Brush Co 360‐9, Universal Airbrush.

## Conflict of Interest

The authors declare no conflict of interest.

## Supporting information

Supplementary

Supplementary

Supplementary

Supplementary

Supplementary

Supplementary

Supplementary

Supplementary

Supplementary

Supplementary
