# Supplementary material for: An Aqueous Composition for Lubricant‐Free, Robust, Slippery, Transparent Coatings on Diverse Substrates
Source: Glob Chall. 2018 Jan 2;2(2):1700097. doi: 10.1002/gch2.201700097 (PMC6607131; doi:10.1002/gch2.201700097)
Supplement: Supplementary file 1 — Supplementary [file GCH2-2-1700097-s002.pdf]

Copyright WILEY-VCH Verlag GmbH & Co. KGaA, 69469 Weinheim, Germany, 2017.

Supporting Information

for *Global Challenges,* DOI: 10.1002/gch2.201700097

An Aqueous Composition for Lubricant-Free, Robust, Slippery, Transparent Coatings on Diverse Substrates

*Avijit Baidya, Sarit Kumar Das, and Thalappil Pradeep**

Copyright WILEY-VCH Verlag GmbH & Co. KGaA, 69469 Weinheim, Germany, 2016.

Supporting Information

An aqueous composition for lubricant free, robust, slippery, transparent coatings on diverse substrates

*Avijit Baidya[a,b], Sarit kumar Das[b] and Thalappil Pradeep[a,]**

Supporting Information Figures

Figure No.	Description	Page No.
Figure S1	EDAX mapping of the coating	2
Figure S2	Powder XRD of the coating material	2
Figure S3	Invisible water barrier	3
Figure S4	Wettabiliy of the coated glass surface after different mechanical tests (20 cycles)	3
Figure S5	Transparency test for mechanically tested surface	4
Figure S6	Universality of the coating	4
Figure S7	Physical appearance of different substrates before and after coating	5
Figure S8	Repellent nature of the coating (on wood and glass) towards oil and emulsion	5

Supporting Videos

Video No.	Description
Video S1	Tansparency experiment
Video S2	Extent of slipperiness
Video S3	Invisible water mountain (top view)
Video S4	Invisible water mountain (side view)
Video S5	Effect of High temperture (200 ºC for 4 h)
Video S6	Effect of low temperture, frost (-80 ºC for 8 h)
Video S7	Effect of aqua regia
Video S8	Behavior of oil and emulsion on coating (glass & wood)
Video S9	Effect of aqua regia on coated metal surface

Supporting Information 1

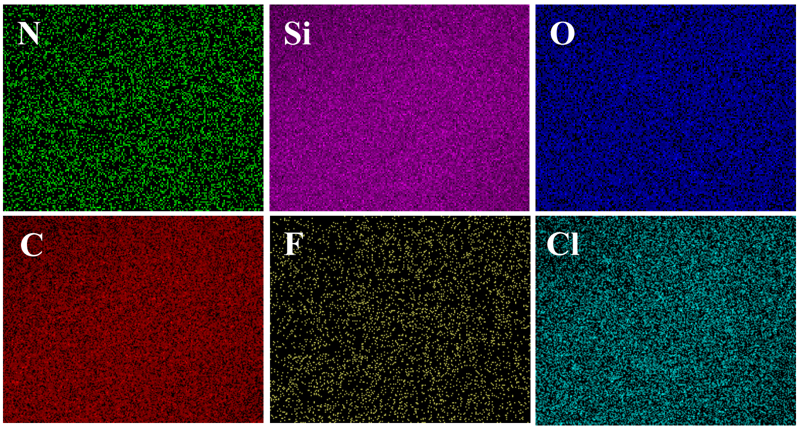

Elem	At %
C	55.87
N	11.30
O	21.91
F	0.20
Si	10.51
Cl	0.22
Total	100.00

Figure S1: SEM EDAX mapping and quantitative distribution of elements.

Supporting Information 2

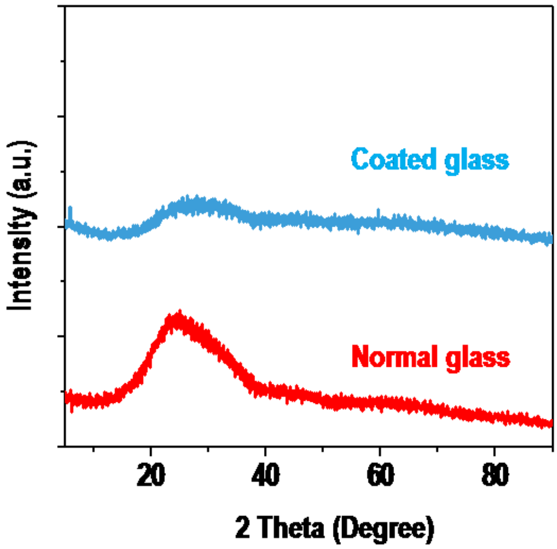

Figure S2: Powder XRD: Amorphous structure of glass is unchanged with coating.

Supporting Information 3

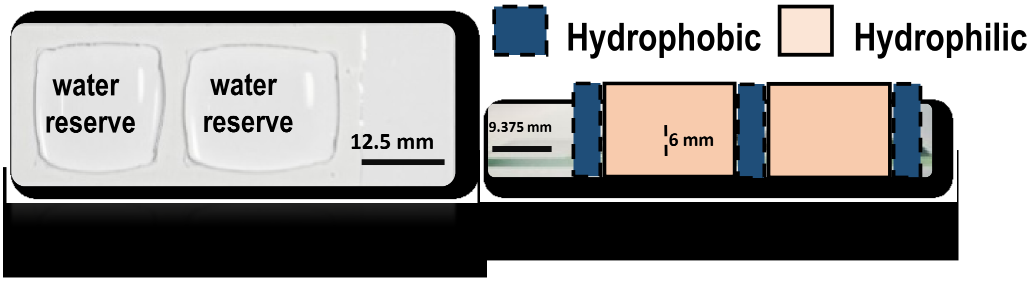

Figure 3: Designed invisible water barrier holds up to 4 mm height of water in air. The top and side view of the water mountain.

Supporting Information 4

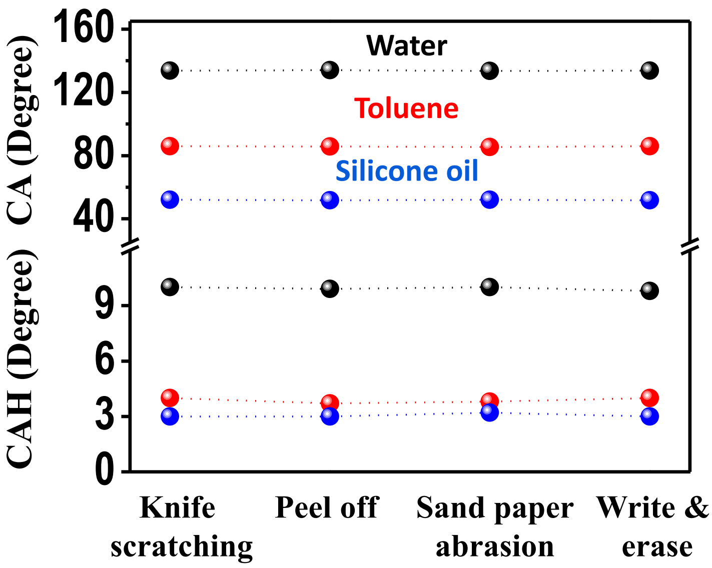

Figure S4: Wettabiliy of the coated glass surface after different mechanical tests (20 cycles).

Supporting Information 5

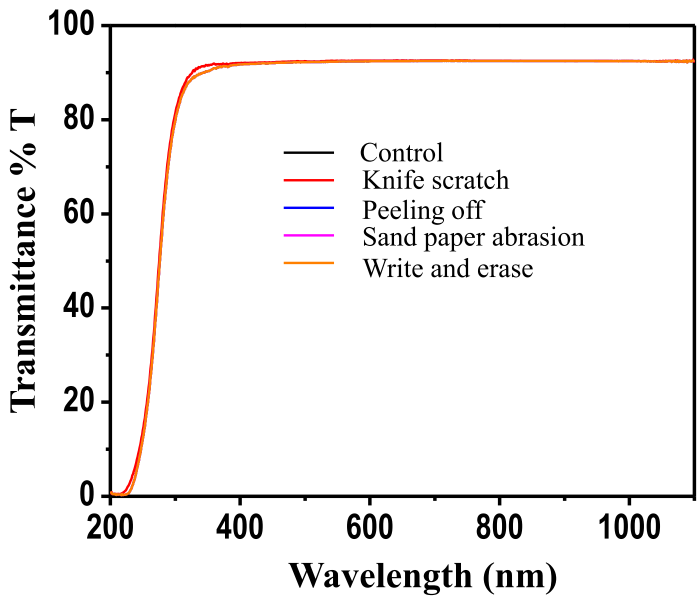

Figure S5: Transparency of the coated glass after different mechanical tests (20 cycles). The graph shows 99.6 % transmittance of the treated substrates with respect to the untreated control.

Supporting Information 6

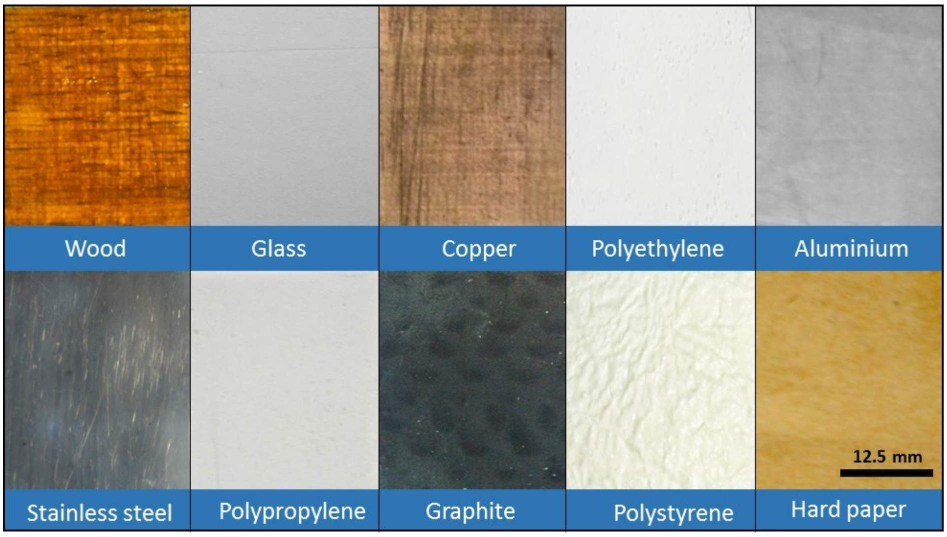

Figure S6: Universality of the coating**.** The coatings can be universally applied to all substrates from wood to plastic and the appearance of the substrate remains unchanged even after the coating.

Supporting Information 7

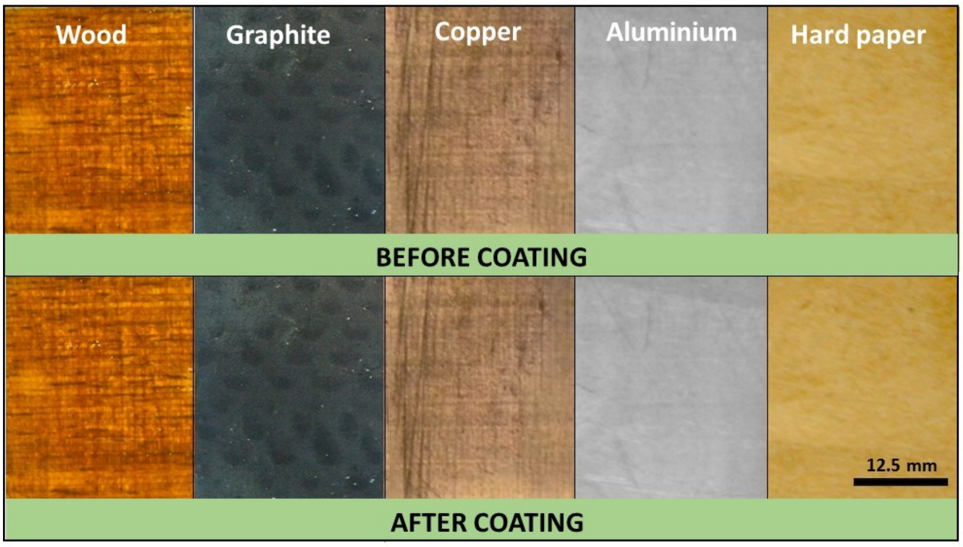

Figure S7: Physical appearance of different substrates before and after coating. There was no change in color in wood as there was no temperature fluctuation (see S8, where there was a color change).

Supporting Information 8

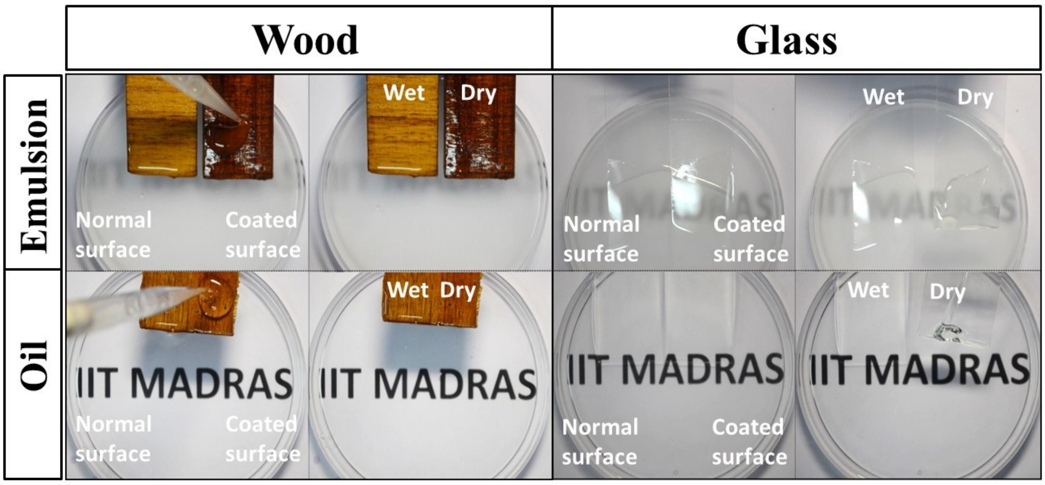

Figure S8: Oil (viscous oil and emulsion) repellent slippery nature of the coating (on wood and glass). Change in color and contrast of the wood is because of temperature fluctuations during the sample preparation. See Figure S7 for a surface without change in color after coating.
